# Evaluation of a standardized test protocol to measure wheelchair-specific anaerobic and aerobic exercise capacity in healthy novices on an instrumented roller ergometer

**DOI:** 10.1371/journal.pone.0274255

**Published:** 2022-09-06

**Authors:** Rowie J. F. Janssen, Riemer J. K. Vegter, Han Houdijk, Lucas H. V. Van der Woude, Sonja de Groot

**Affiliations:** 1 Center for Human Movement Sciences, University Medical Center Groningen, University of Groningen, Groningen, The Netherlands; 2 Peter Harrison Centre for Disability Sports, School of Sport Exercise and Health Sciences, Loughborough University, Loughborough, United Kingdom; 3 Center for Rehabilitation, University Medical Center Groningen, Groningen, The Netherlands; 4 Department of Human Movement Sciences, Faculty of Behavioural and Movement Sciences, Vrije Universiteit Amsterdam, Amsterdam, The Netherlands; 5 Amsterdam Rehabilitation Research Center Reade, Amsterdam, The Netherlands; Sport Sciences School of Rio Maior - Politechnic Institute of Santarem, PORTUGAL

## Abstract

This study aims to evaluate whether a test protocol with standardized and individualized resistance settings leads to valid wheelchair Wingate tests (WAnT) and graded exercise tests (GXT) in healthy novices. Twenty able-bodied individuals (10M/10F, age 23 ± 2 years, body mass 72 ± 11 kg) performed an isometric strength test, sprint test, WAnT and GXT on a wheelchair ergometer. Using a previously developed set of regression equations, individuals’ isometric strength outcome was used to estimate the WAnT result (P30_est_), from which an effective individual WAnT resistance was derived. The subsequently measured WAnT outcome (P30_meas_) was used to estimate the GXT outcome (POpeak_est_) and to scale the individual GXT resistance steps. Estimated and measured outcomes were compared. The WAnT protocol was considered valid when maximal velocity did not exceed 3 m·s^-1^; the GXT protocol was considered valid when test duration was 8–12 min. P30_est_ did not significantly differ from P30_meas_, while one participant did not have a valid WanT, as maximal velocity exceeded 3 m·s^-1^. POpeak_est_ was 10% higher than POpeak_meas_, and six participants did not reach a valid GXT: five participants had a test duration under 8 min and one participant over 12 min. The isometric strength test can be used to individually scale the WAnT protocol. The WAnT outcome scaled the protocol for the GXT less accurately, resulting in mostly shorter-than-desired test durations. In conclusion, the evaluated standardized and individualized test protocol was valid for the WAnT but less valid for the GXT among a group of novices. Before implementing the standardized individual test protocol on a broader scale, e.g. among paralympic athletes, it should be evaluated among different athletic wheelchair-dependent populations.

## 1 Introduction

Participation in wheelchair sports and the interest in it have developed strongly over the past decades, leading to a more professional approach in wheeled sports and clinical wheelchair practice [1, 2]. To optimize wheeling performance, exercise capacity tests are becoming a central topic of interest for sport and rehabilitation scientists, coaches and athletes. The most commonly used performance indicators of wheelchair-specific exercise capacity are anaerobic and aerobic capacity [3]. Anaerobic capacity is important when brief bouts of maximal efforts are required [3, 4]. Aerobic capacity reflects the capacities of the cardiovascular and respiratory systems to supply oxygen to the exercising muscles in more prolonged exercise periods [3, 4].

Wheelchair-specific anaerobic capacity can be assessed with a Wingate test (WAnT), which is a 30 s sprint test against a predetermined high resistance [5]. The proper resistance should be set individually. If the resistance is set too high, the athlete cannot accelerate the wheelchair, if it is too low it may lead to hand velocities that exceed the level of proper coordination of hand-to-rim interactions (i.e. > 3.0m·s^-1^). Both non-optimal conditions limit power production as muscles must act at excessive/insufficient force levels [6, 7]. Wheelchair-specific aerobic capacity can be assessed through a graded exercise test (GXT), which ideally lasts 8–12 min [8]. Shorter protocols with higher resistances tend to induce muscle fatigue, longer protocols with lower resistances will cause higher body temperature, dehydration, discomfort and/or ventilatory muscle fatigue [8].

A recent literature review of studies examining WAnTs and GXTs in wheelchair athletes [9] showed that resistance settings differed among the studies and were often based on the expertise of the test leader and/or a priori knowledge on the evaluated athletes. These protocol settings are difficult to reproduce, hampering comparison among studies. Given the considerable heterogeneity among wheelchair athletes’ exercise capacities and the intention to compare between and among athletes, there is a need for standardized and individualized protocols for exercise testing. Ideally, such protocols would use individual resistance settings that lead to optimal velocities in the WAnT and optimal durations in the GXT [7, 8].

A possible solution toward a standardized and individualized testing protocol may be found in a study that showed strong associations between upper-body isometric strength and anaerobic and aerobic power in male wheelchair users with a spinal cord injury [10]. A set of regression equations was developed between the main outcomes of a wheelchair-specific isometric strength test (F_iso_), a WAnT (P30) and a GXT (POpeak). In contrast with the WAnT and GXT protocols, wheelchair-specific isometric strength can be measured in a relatively simple and standardized way in different individuals using identical experimental settings. The participant–sitting in their handrim wheelchair–exerts maximal force for 5 s on top of the blocked handrims (this is feasible either on an ergometer [11–14] or an alternative set up [12]). Using the previously established regression equation (P30_est_ = 0.51 * F_iso_− 0.18 [10]), the individual P30 can be estimated from the just-measured F_iso_, then an effective WAnT resistance can be determined. Similarly, based on the previously established association between P30 and POpeak (POpeak_est_ = 0.67 * P30 + 0.11 [10]), individual POpeak can be estimated from the just-measured P30, then effective resistance settings for the GXT can be determined. Ideally, with this standardized and individualized procedure everyone could utilize these equations in the same way. How accurate these equations are in different participant groups (e.g. able-bodied subjects, athletes) needs further consideration.

A few studies implemented this standardized and individualized test protocol in a rehabilitation population and/or able-bodied individuals [11–14]. However, these tests were performed on an integrated computer-controlled wheelchair simulator, which is accommodated with a seat and handrim that can be adjusted to an individual user only to a limited degree [15]. While wheelchair athletes have highly individualized wheelchairs, performance tests are preferably done in their own sports wheelchair, which in turn is possible on an instrumented wheelchair roller ergometer (or treadmill) [16]. However, at high forces, the isometric strength test might not be feasible on a wheelchair roller ergometer, as at some point the wheels of the wheelchair may slip on the rollers of the ergometer or the electromechanical break may fail. A 10 s sprint test is therefore included in the current test protocol as a possible alternative for the estimation of anaerobic capacity.

Besides these methodological considerations, none of these earlier studies [11–14] verified whether the protocol led to outcomes close to the individually estimated values or to valid WAnT and GXT resistance settings. As stated, for the WAnT the participant should be able to accelerate against a sufficiently high resistance and maximal velocity should not exceed 3 m·s^-1^ [17–19]. The GXT protocol is deemed valid when test duration is 8–12 min [8]. Secondary criteria are a peak heart rate (HR_peak_) over 95% of the age-predicted HR (200-age) and a rate of perceived exertion (RPE) over 8 [20]. As a first exploration of the potential of this test protocol and before using it in an athletic wheelchair population, we included an able-bodied population.

The main aim of this study was to evaluate a standardized and individualized test protocol to measure wheelchair-specific anaerobic and aerobic exercise capacity among a group of healthy novices on an instrumented dual roller wheelchair ergometer. First, we evaluated whether the measured outcomes for the WAnT and GXT were close to the estimated values. Next we reconstructed the regression equations, based on our population of novices, between isometric strength and anaerobic power and between anaerobic and aerobic power. Because of the potential methodological limitations of the isometric strength test, we additionally estimated anaerobic power based on the 10 s sprint test outcome. Last, the validity of the WAnT and GXT outcomes were evaluated against the aforementioned criteria.

## 2 Materials and methods

### 2.1 Participants

Twenty able-bodied participants (10M/10F) were included. Inclusion criteria were age 18–30 years and the absence of medical contraindications for exercise according to the Physical Activity Readiness Questionnaire [21]. To allow for sufficient heterogeneity of the study group, no further inclusion criteria were set. Participants had a mean age of 23 ± 2 years, a body mass of 72 ± 11 kg and a height of 1.78 ± 0.10 m, and practiced different sports (e.g. climbing, athletics) for 5 ± 3 hours per week on average (Table 1). They had no prior experience in wheelchair propulsion. On the day before testing, participants were asked to refrain from heavy sports activities and to maintain a normal sleeping pattern. Participants voluntarily participated in the study after signing an informed consent. The local ethics committee of the Center for Human Movement Sciences, University Medical Center Groningen, University of Groningen, The Netherlands approved the study protocol (202000455).

**Table 1:** Participant characteristics together with the individual and group average test results for the isometric strength test, sprint test, wingate test and graded exercise test.

Participant						Isometric		Sprint test						Wingate test									Graded Exercise test								
Number	Gender [F/M]	Age [yrs]	Body mass [kg]	Height [cm]	TH/week [hrs]	F_iso_ [N]	F_iso_ [N·kg^-1^]	v_mean_ [m·s^-1^]	v_max_ [m·s^-1^]	PO_mean_ [W]	PO_mean_ [W·kg^-1^]	PO_max_ [W]	PO_max_ [W·kg^-1^]	P30_est_ [W]	P30_meas_ [W]	Δ P30_est-meas_ [%]	P30_meas_ [W·kg^-1^]	P5 [W]	PO_max_ [W]	RF [%]	v_mean_ [m·s^-1^]	v_max_ [m·s^-1^]	POpeak_est_ [W]	POpeak_meas_ [W]	Δ POpeak_est-meas_ [%]	POpeak_meas_ [W·kg^-1^]	Duration [s]	HR_peak_ [bpm]	RPE_c_	RPE_p_	RPE
**1**	F	22	63	163	4	129	2.1	2.1	2.7	45	0.7	350	5.6	55	55	1	0.9	63	366	17	1.9	2.2	43	41	-5	0.7	600	169	7	9	8
**2**	F	23	68	170	3	158	2.3	2.1	2.9	54	0.8	500	7.4	68	63	-8	0.9	81	473	49	1.8	2.3	50	38	-23	0.6	420	168	8	10	9
**3**	F	24	68	172	2	178	2.6	2.4	3.1	61	0.9	579	8.5	79	79	-1	1.2	96	534	33	1.9	2.4	61	44	-28	0.7	450	181	7	9	8
**4**	M	23	94	189	6	344	3.7	2.8	3.3	85	0.9	1092	11.6	160	125	-22	1.3	166	857	33	1.6	2.2	95	81	-15	0.9	540	178	7	8	8
**5**	M	28	76	176	5	**-**	**-**	3.0	3.5	84	1.1	792	10.4	-	136	-	1.8	176	864	40	1.7	2.4	-	-	-	-	-	-	-	-	-
**6**	F	21	71	184	8	152	2.1	2.5	3.4	69	1.0	535	7.5	65	67	4	1.0	87	525	49	2.0	2.5	53	55	4	0.8	600	166	10	9	10
**7**	F	24	67	172	4	202	3.0	2.3	2.7	48	0.7	508	7.6	91	78	-14	1.2	112	466	48	1.7	2.2	-	-	-	-	-	-	-	-	-
**8**	M	24	75	178	3	220	2.9	2.5	3.1	62	0.8	612	8.2	98	91	-7	1.2	121	656	48	1.8	2.5	70	57	-18	0.8	480	186	6	8	7
**9**	F	19	62	170	6	139	2.2	2.8	3.3	61	1.0	429	6.9	60	59	-1	1.0	98	438	58	1.9	2.6	47	44	-7	0.7	600	187	5	8	7
**10**	M	23	75	186	4	293	3.9	3.1	4.3	104	1.4	1027	13.7	136	172	26	2.3	162	990	15	2.5	3.0	123	113	-8	1.5	540	192	7	9	8
**11**	F	23	56	164	3	153	2.7	2.6	3.3	55	1.0	399	7.1	68	66	-2	1.2	86	380	40	1.9	2.3	50	56	11	1.0	720	187	7	8	8
**12**	F	21	60	169	12	216	3.6	2.8	3.2	57	0.9	376	6.3	99	87	-12	1.5	106	417	37	1.8	2.2	65	64	-2	1.1	660	175	7	10	9
**13**	M	23	96	191	12	341	3.6	3.0	3.8	117	1.2	1077	11.2	156	162	4	1.7	224	1058	47	2.0	2.7	-	-	-	-	-	-	-	-	-
**14**	M	24	67	188	8	268	4.0	3.0	3.8	81	1.2	759	11.3	124	128	4	1.9	169	768	46	2.1	2.6	93	80	-14	1.2	540	187	6	9	8
**15**	M	25	81	188	3	184	2.3	2.2	2.8	54	0.7	581	7.2	79	68	-13	0.9	81	480	34	1.7	2.0	58	53	-9	0.7	570	186	8	7	8
**16**	F	20	55	163	4	132	2.4	2.1	2.9	43	0.8	355	6.5	58	58	-1	1.1	78	446	42	1.9	2.4	44	42	-5	0.8	540	181	8	9	9
**17**	M	22	80	191	5	322	4.0	3.0	3.8	94	1.2	789	9.9	150	150	0	1.9	161	736	23	2.0	2.5	110	83	-24	1.0	450	183	8	10	9
**18**	M	25	89	188	2	179	2.0	2.2	2.9	67	0.8	701	7.9	75	78	4	0.9	98	711	37	2.0	2.2	62	82	32	0.9	840	189	7	8	8
**19**	F	20	77	188	8	165	2.1	2.4	3.0	62	0.8	448	5.8	70	84	21	1.1	108	566	38	2.3	2.7	64	48	-25	0.6	420	171	8	9	9
**20**	M	23	69	179	5	293	4.3	3.0	3.8	87	1.3	670	9.7	136	125	-8	1.8	148	626	37	1.8	2.4	91	57	-37	0.8	420	166	7	9	8
*Mean*	**-**	**23**	**72**	**178**	**5**	**214**	**2.9**	**2.6**	**3.3**	**69**	**1.0**	**629**	**8.5**	**96**	**97**	**-1**	**1.3**	**121**	**618**	**38**	**1.9**	**2.4**	**69**	**61**	**-10**	**0.9**	**552**	**180**	**7**	**9**	**8**
*SD*	**-**	**2**	**11**	**10**	**3**	**73**	**0.8**	**0.3**	**0.4**	**20**	**0.2**	**234**	**2.2**	**36**	**37**	**11**	**0.4**	**43**	**205**	**11**	**0.2**	**0.2**	**24**	**20**	**16**	**0.2**	**114**	**9**	**1**	**1**	**1**

Values that do not meet the assumptions of a valid test or lay outside the 20%-boundaries are highlighted in dark gray.

### 2.2 Measurement set-up

Tests were conducted at two locations (University Medical Center Groningen & Reade Rehabilitation center Amsterdam) by two test leaders between March and June 2021. Both test leaders followed identical protocols, and to avoid differences between the test leaders pilot participants were tested together before starting the actual testing period. On both locations, the room temperature was held constant at 20° and humidity was 50% for all tests. Half of the tests were performed in the morning and the other half in the afternoon.

**Fig 1:**
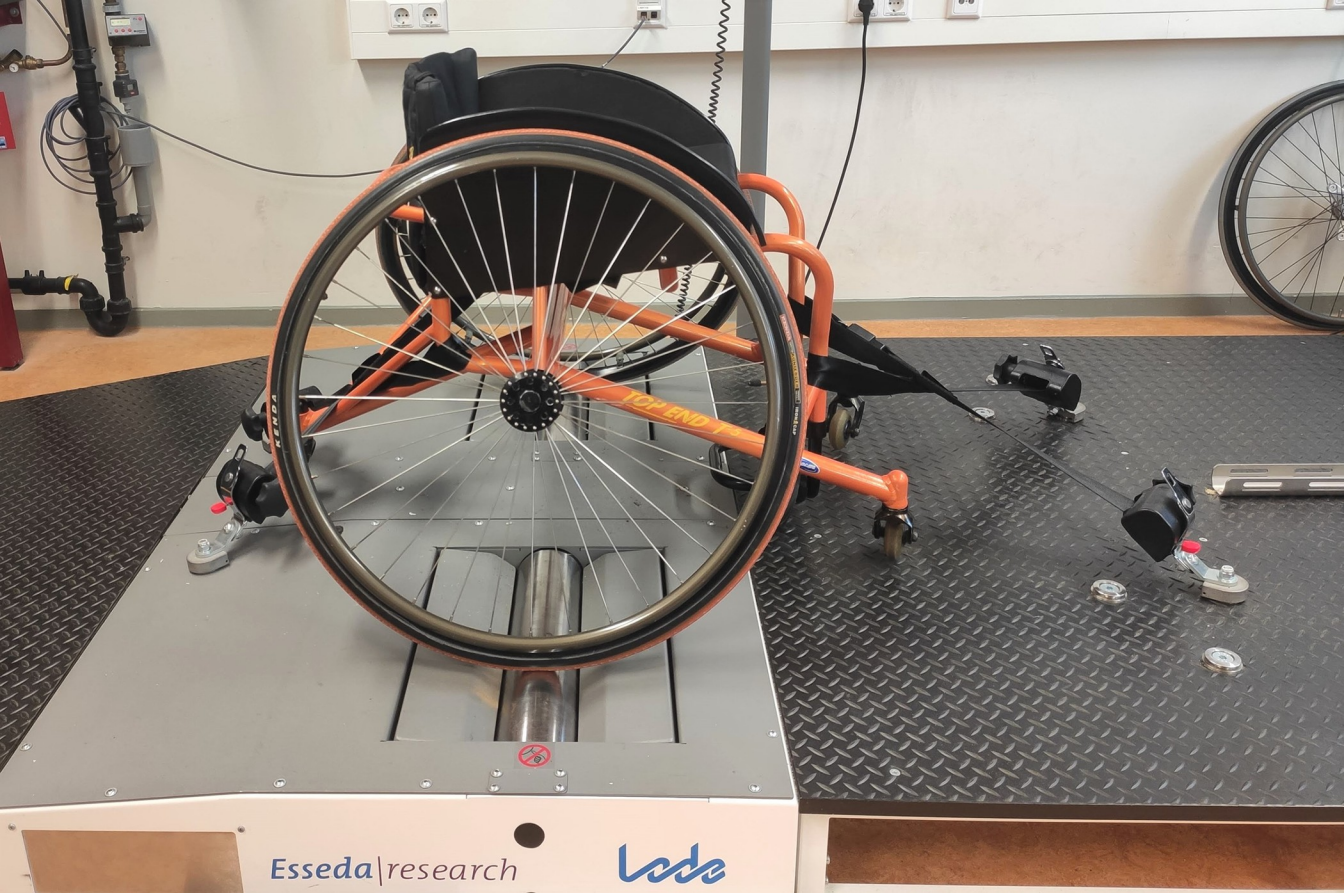
The Esseda wheelchair roller ergometer with the wheelchair used in Groningen.

The computer-controlled Esseda wheelchair roller ergometer was available at both locations and used for testing (Fig 1, Lode BV, Groningen, The Netherlands). This commercial wheelchair ergometer allows for accurate individual simulation of wheelchair propulsion, inertia and resistance, while allowing for accurate measurements (100 Hz) of both left- and right-hand propulsion characteristics [22]. Before each test, the ergometer was calibrated to account for static and dynamic friction for each individual-wheelchair combination [22]. In Groningen, a tennis wheelchair (Double Performance BV, Gouda, The Netherlands) was used with a mass of 10 kg, a wheel radius of 0.34 m and a rim radius of 0.31 m. In Amsterdam, a rugby wheelchair (Top End Invacare, Ede, The Netherlands) was used with a mass of 14.5 kg, a wheel radius of 0.31 m and a rim radius of 0.28 m. The tires of both wheelchairs were inflated up to the recommended pressure, 7 Bar.

All analyses were done with a custom-written Python script (Work lab package, DOI 10.5281/zenodo.3268671). Torque and velocity data were directly derived from the wheelchair ergometer (100 Hz) and filtered with a fourth-order low-pass Butterworth filter (cut-off frequency 10 Hz). The effective force on the handrims (F) at each side was calculated from the measured torque (M) and rim radius (r_r_):

$$F\;[N]=M\;[Nm]*{r_{r}}^{-1}\;[m]$$ [Eq 1]

The power output (PO) at each side was calculated from the measured torque (M), wheel radius (r_w_) and wheel velocity (v):

$$PO\;[W]=M\;[Nm]*{r_{w}}^{-1}\;[m]*v\;[m\cdot s^{-1}]$$ [Eq 2]

### 2.3 Test protocols

**Fig 2:**
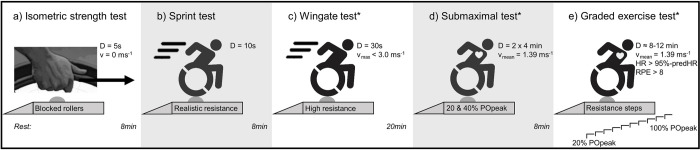
Schematic overview of the experimental protocol. Set-up for the five tests. D = duration, v = velocity, HR = heart rate, RPE = rate of perceived exertion, * = resistance is individually determined.

In the laboratory, participants were asked to perform five tests in a fixed sequence with standardized rest periods in-between (Fig 2). First an isometric strength test was performed, followed by two 10 s sprint tests, a 30 s WAnT, a submaximal test of two 4-min low-intensity bouts at 1.39 m·s^-1^, and last, a GXT. Verbal encouragement was provided throughout each test. Before starting with the test battery, a 3-min warm-up at 1.39 m·s^-1^ was performed for purposes of familiarization with ergometer-based handrim wheelchair propulsion and the testing set-up. The submaximal test was additionally performed to gain insights into the propulsion technique, but falls beyond the primary scope of the current study and will not be presented here.

#### 2.3.1 Isometric strength test

**Fig 3:**
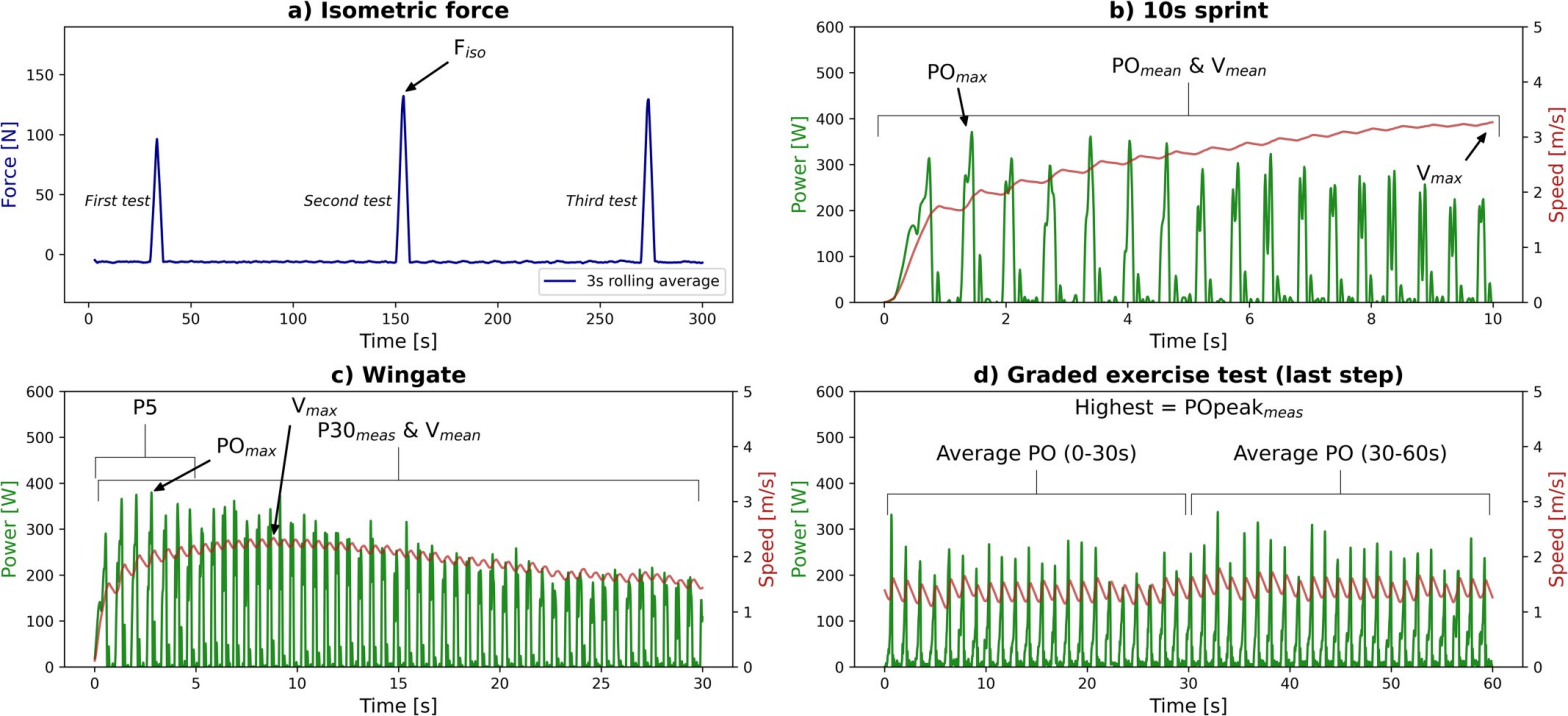
Extracted variables for the a) isometric strength test, b) sprint test, c) Wingate test and d) graded exercise test. Strength (F) is the average of both arms (in blue); power output (PO) is the sum of both arms (in green); velocity (v) is the average of both arms (in red).

Participants–sitting in the wheelchair–exerted three times, with 2 min rest in-between tests, maximal force with both hands for 5 s on the top dead center along the tangential direction of the handrim (Fig 2A). Maximal isometric strength (F_iso_) was defined as the highest mean F (averaged over left and right arms) over a 3 s rolling average (Fig 3A).

#### 2.3.2 Sprint test

Participants performed two 10 s sprints from stationary start, with 2-min rest in-between (Fig 2B). Resistance was set to that of a gym court (rolling resistance coefficient (μ) = 0.012) [22]. Mean and maximal sprint power output and velocity (PO_mean_ [W], PO_max_ [W], v_mean_ [m·s^-1^], v_max_ [m·s^-1^]) were defined as the average PO and v over 10 s and as the one sample highest peak, respectively (Fig 3B). PO_mean_ and PO_max_ were defined as the sum of both arms, v_mean_ and v_max_ as the average of both arms. The sprint with the highest v_max_ was chosen for further analysis.

#### 2.3.3 Wingate test (protocol set up)

Participants performed a 30 s sprint test at a high individualized resistance, calculated from the estimated outcome, from a stationary start (Fig 2C). Estimated anaerobic mean PO (sum of both arms) over 30 s (P30_est_), as calculated from F_iso_ (averaged over left and right arms) according to the regression equation from earlier research [10]:

$${P30}_{est}\;[{W\cdot kg}^{-1}]=0.51*F_{iso}{\;[W\cdot kg}^{-1}]-0.18$$ [Eq 3]

Absolute P30_est_ was acquired by multiplying P30_est_ by the mass of the user:

$${P30}_{est}\;[W]={P30}_{est}\;[{W\cdot kg}^{-1}]*m_{user}\;[kg]$$ [Eq 4]

To stay below 3 m·s^-1^ during the Wingate, the resistance coefficient was calculated from the estimated P30_est_ at a mean velocity of 2 m·s^-1^. From the P30_est_ and the v_mean_, the required resistance coefficient (R) was determined relative to the total mass of the participant and the wheelchair (m_total_):

$$R\;[\mu ]={P30}_{est}\;[W]*{v_{mean}}^{-1}\;[{m\cdot s}^{-1}]*{m_{total}}^{-1}\;[N]$$ [Eq 5]

#### 2.3.4 Wingate test (outcomes)

The P30 measured during the test (P30_meas_) was calculated as the mean PO over 30 s, P5 as the highest PO over successive 5 s intervals and POmax as the one sample highest peak (Fig 3C). All PO outcomes are the sum of both arms. Rate of fatigue (RF) was calculated as: (P5_start_−P5_end_) / P5_start_ * 100%. Average velocity (v_mean_) was defined as the average velocity over 30 s and maximal velocity (v_max_) as the highest peak. v_mean_ and v_max_ are the average of both arms.

#### 2.3.5 Graded exercise test (protocol set-up)

Participants performed a GXT to exhaustion (Fig 2E). Resistance increased every minute, while maintaining a constant velocity of 1.39 m·s^-1^ [13]. A computer screen in front of the participant provided visual feedback on the actual left and right speed and the set target speed. Estimated aerobic power (POpeak_est_) was calculated from P30_meas_ (both as the sum of both arms) according to the following regression equation [10]:

$${POpeak}_{est}\;[{W\cdot kg}^{-1}]=0.67*{P30}_{meas}\;[{W\cdot kg}^{-1}]+0.11$$ [Eq 6]

By using Eqs 4 and 5, with POpeak_est_ replacing P30_est_, the initial resistance was set equal to 20% of POpeak_est_. In every subsequent one-minute step the resistance increased by 10% of the difference between starting load and POpeak_est_, so that exhaustion and the actual POpeak would be reached at around 10 min. During the test, heart rate (HR) was recorded using a Garmin HR monitor (Kansas City, MO, USA) or Polar HR monitor (Kempele, Finland). The test was terminated when the participant could no longer maintain the required velocity.

#### 2.3.6 Graded exercise test (outcomes)

POpeak_meas_ was calculated as the highest mean PO (sum of both arms) achieved over 30 s (Fig 3D) [23]. HR_peak_ was the one sample highest peak. At the end of the test, peripheral and central RPE (RPE_p_ and RPE_c_) were asked on a scale from 1 to 10 [24]. RPE_p_ and RPE_c_ refer to the RPE in the peripheral exercising muscles and the cardiorespiratory system, respectively. The average of RPE_p_ and RPE_c_ together form the overall RPE [25].

### 2.4 Statistical analysis

The outcomes for the WAnT (P30) and GXT (POpeak) were compared with the estimated values and tests were evaluated on the preset validity criteria. The Shapiro-Wilk test showed that differences between P30_meas_ and P30_est_ were not normally distributed (p = 0.04), while differences between POpeak_meas_ and POpeak_est_ were (p = 0.83). Hence a Wilcoxon signed rank test and a paired t-test were conducted to check for systematic differences between the measured and estimated values for P30 and POpeak, respectively. Significance was set at p < 0.05. If a significant effect was found, post-hoc statistical power analyses were performed with G*Power (version 3.1.9.4) [26]. To gain more insight into the deviation between the individual estimated and measured values, scatterplots were made with 20% deviation boundaries. These boundaries were based on a test duration of 10 ± 2 min (= 20%) to reach POpeak in the GXT [8], and P30 follows these 20% boundaries.

Non-parametric Theil-Sen regression procedures [27] were used to reconstruct the regression equations to estimate the WAnT and GXT results–P30 and POpeak, respectively. The results of the isometric strength test and the sprint test were used as independent variables to estimate P30; the WAnT result was used to estimate POpeak. Inclusion was at the 0.05 level. To compare our results to regression equations from previous studies [10, 28], all variables in the regression were expressed per kilogram body mass. Python 3.8 (Python Software Foundation) was used for all analyses.

## 3 Results

### 3.1 Main test results

All individual outcomes are shown in Table 1, as are group means and standard deviations for the four subsequent tests (isometric strength test, sprint test, WAnT and GXT). Values that do not meet the assumptions of a valid test or lay outside the 20% boundaries are highlighted in dark gray.

#### 3.1.1 Isometric strength test

Isometric strength could be measured in 19 out of 20 participants, and the average F_iso_ was 214 ± 73 N. The strongest participant pushed through the brakes of the ergometer and two participants experienced slipping on the rollers of the ergometer. The slipping was minimal and F_iso_ could still be calculated, while for the participant that pushed through the brakes this was not possible.

#### 3.1.2 Sprint test

Sprint test data were collected for 37 out of 40 sprints. A technical problem occurred when exporting the ergometer data for three participants during one of their sprints. Average v_mean_ and v_max_ were 2.6 ± 0.3 and 3.3 ± 0.4 m·s^-1^, respectively, average PO_mean_ and PO_max_ during the sprint test were 69 ± 20 and 629 ± 234 W, respectively.

#### 3.1.3 Wingate test

**Fig 4:**
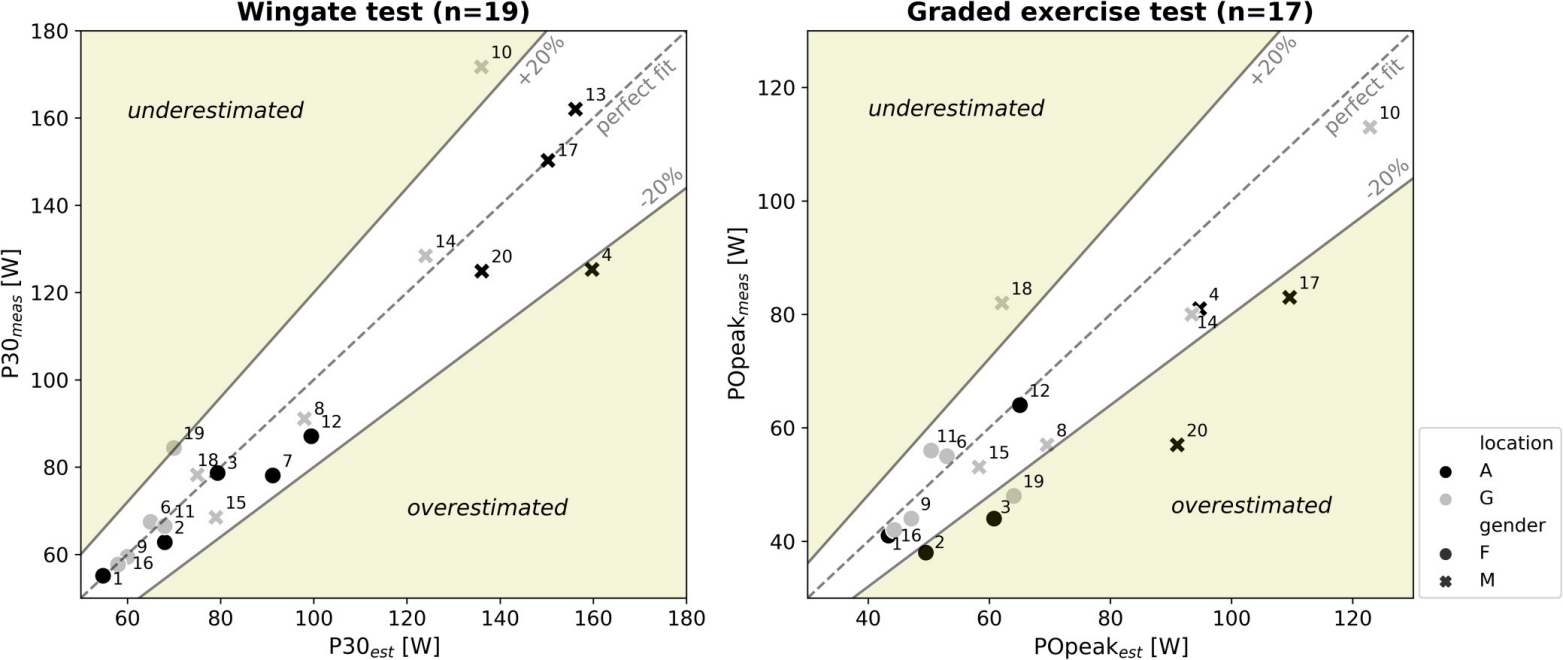
Comparison of the individual estimated P30 (left graph) and POpeak (right graph) with the actual measured P30 and POpeak. The number attached to the symbol is the same as participant numbers in Table 1. Dotted lines correspond w a perfect fit and solid lines represent 20% boundaries. A = measured in Amsterdam, G = measured in Groningen, F = female, M = male.

All WAnTs were successfully performed. P30_meas_ did not significantly differ from P30_est_ (p = 0.418). Three participants, however, had a difference of over 20% (-22, +21 and +26%; Fig 4). v_max_ was on average 2.4 ± 0.2 m·s^-1^, while v_max_ of one participant was 3.01 m·s^-1^ and considered non-valid.

#### 3.1.4 Graded exercise test

A technical problem occurred during two GXTs and one participant had to stop because of blisters, so 17 GXTs were analyzed. POpeak_est_ was significantly higher than POpeak_meas_ (difference 10 ± 16%, p = 0.015, post-hoc statistical power 0.74). Six participants had a difference of over 20% (range: -37 to +32%; Fig 4). Five participants had a test duration under 8 min and one participant over 12 min, so six tests were considered non-valid. Two participants did not reach the 95% age-predicted HR_peak_ and two others rated an overall RPE under 8.

### 3.2 Theil-Sen regression results

The results from the Theil-Sen regression procedures are reported in Table 2. Among the parameters to estimate P30, F_iso_ showed the highest explained variance (R^2^ = 0.82), followed by PO_mean_ from the sprint test (R^2^ = 0.80). Estimation of POpeak_meas_ from P30_meas_ resulted in an explained variance of 0.67. Although the intercepts of the equations to estimate P30 from F_iso_ and P30 from PO_max_ were not significantly different from zero, they were included in the equations, since these equations best described the associations found in this study.

**Table 2:** Results from the Theil-Sen regression of the current study and from other studies that investigated the same wheelchair-specific associations.

Dependent variable	Independent variable	Regression equation	R^2^	p-value (a, b)	n
**Able-bodied male and female participants (data current experiment)**					
WAnT -P30 [W·kg^-1^]	Strength-F_iso_ [N·kg^-1^]	P30 = 0.43 * F_iso_− 0.01	0.82	< 0.01, 0.39	19
WAnT-P30 [W·kg^-1^]	Sprint-v_mean_ [m·s^-1^]	P30 = 1.08 * v_mean_ -1.47	0.77	< 0.01, < 0.01	20
WAnT-P30 [W·kg^-1^]	Sprint-v_max_ [m·s^-1^]	P30 = 0.88 * v_max_ -1.54	0.79	< 0.01, < 0.01	20
WAnT-P30 [W·kg^-1^]	Sprint-PO_mean_ [W·kg^-1^]	P30 = 1.83 * PO_mean_—0.38	0.80	< 0.01, < 0.01	20
WAnT-P30 [W·kg^-1^]	Sprint-PO_max_ [W·kg^-1^]	P30 = 0.14 * PO_max_ + 0.08	0.67	< 0.01, 0.92	20
GXT-POpeak [W·kg^-1^]	WAnT-P30 [W·kg^-1^]	POpeak = 0.46 * P30 + 0.25	0.67	< 0.01, < 0.01	17
**Inactive men with a spinal cord injury (age = 34 ± 12, body mass = 79 ± 16, TH/week = 3 ± 3)** [10]					
WAnT-P30 [W·kg^-1^]	Strength-F_iso_ [N·kg^-1^]	P30 = 0.51 * F_iso_—0.18	0.75	<0.01, 0.15	44
GXT-POpeak [W·kg^-1^]	WAnT-P30 [W·kg^-1^]	POpeak = 0.67 * P30 + 0.11	0.81	<0.01, 0.09	44
**Male wheelchair track athletes (age = 27 ± 5, body mass = 60 ± 12, TH/week = 16 ± 7)** [28]					
GXT-POpeak [W·kg^-1^]	WAnT-P30 [W·kg^-1^]	POpeak = 0.75 * P30 + 0.03	0.77	-	23

R^2^ = explained variance, a = slope of regression equation, b = intercept of regression equation.

## 4 Discussion

This study evaluated a standardized and individualized test protocol to measure wheelchair-specific anaerobic and aerobic exercise capacity in healthy novices on an instrumented roller ergometer. The isometric strength test estimated the 30 s WAnT adequately. Only one out of twenty participants just exceeded the upper speed limit, with a v_max_ of 3.01 m·s^-1^. The isometric strength test can therefore be seen as a good-enough indicator of P30 in this healthy population, which allows scaling the protocol to individual participants’ abilities. The results of the WAnT significantly overestimated (10%; POpeak_est_ > POpeak_meas_) the actual outcomes of the GXT, and the GXT protocol was not sufficiently accurate in six out of 17 participants. Compared to the estimation of P30, POpeak was not as well-estimated, resulting in shorter-than-desired test durations.

The measured average F_iso_ (214 N), P30 (97 W) and POpeak (61 W) are slightly lower compared with results from previous research in able-bodied participants who performed the same test battery on a computer-controlled ergometer with fixed wheelchair settings (F_iso_ = 232 N, P30 = 119 W, POpeak = 64 W) [13]. This might be due to differences in gender, as that study only included males and the current study included both males and females. Wheelchair settings may have played another influencing role. Able-bodied individuals served as a first exploration for the potential of the standardized test protocol. Future research should evaluate this test protocol in wheelchair athletes. Isometric strength (averaged over left and right arms) ranges from 129 ± 68 N in trained wheelchair rugby individuals to 287 ± 45 N in elite wheelchair basketball athletes [14, 29]. P30 (sum of both arms) ranges from 23 ± 4 W to 138 ± 24 W and POpeak (sum of both arms) from 22 ± 9 to 117 ± 4 W, all found in different classes of wheelchair racing athletes [18, 28]. The effects of sport discipline, training and disability explain the wide range of test results found among wheelchair athletes, but variation and study differences are also amplified by the use of different methods (e.g. testing device, wheelchair geometry, test protocol) [9]. Nevertheless, the three main outcomes (F_iso_, P30, POpeak) of the current study all seem to fall within the range of results obtained by wheelchair athletes, which seems to enhance the potential of this test protocol studied in wheelchair athletes.

### 4.1 Settings of the wheelchair-specific anaerobic exercise test

Regarding the WAnT, most individuals are spread around the “perfect fit” in Fig 4; the Wilcoxon test showed that the estimated values (P30_est_) did not significantly differ from the measured values (P30_meas_). Some individual variation around the “perfect fit” is expected, as every participant is unique in their responses; some are relatively stronger, others anaerobically more fit or skilled. While all participants could accelerate against the high resistance and only one out of the twenty participants just exceeded the upper speed limit (v_max_ = 3.01 m·s^-1^), we can conclude that WAnT resistance can be individually well-scaled from the isometric strength test in able-bodied individuals.

Some might argue that wheelchair athletes are more skilled and have a better sprint technique, which leads to better results. However, an earlier study showed no differences in P30 during a 30 s sprint on a wheelchair ergometer between able-bodied and wheelchair-dependent individuals [30]. The isometric strength test is a standardized test where technique is expected to have a less significant role. Besides, the original regression equations were based on inactive wheelchair males with spinal cord injuries [10], and although a different group was included in the current study (able-bodied participants), it led to valid resistance settings. This leads to our hypothesis that a valid WAnT resistance can be derived from the F_iso_ in wheelchair athletes.

In our studied population one participant did push fully through the brakes of the ergometer and we had to estimate his anaerobic capacity based on our experience, which is not reproducible. Even higher isometric strengths were found in international wheelchair basketball athletes (287 ± 45 N) [29], therefore the isometric strength test might not be feasible for all wheelchair athletes on a current instrumented wheelchair roller ergometer. In contrast to the isometric strength test, the 10 s sprint test could be performed by all participants. It is also a more familiar task to perform because sprinting is, to varying degrees, important in every wheelchair sport [31], while the isometric strength test may feel unnatural for some. The explained variance to estimate P30 based on PO_mean_ of the sprint test has comparable strength to the equation based on F_iso_ from earlier research [10] (R^2^ = 0.80 vs R^2^ = 0.75; Table 2). However, before adopting it in another study population the validity of the estimated settings based on the PO_mean_ of the 10 s sprint test needs to be evaluated more systematically.

### 4.2 Settings of the wheelchair-specific aerobic exercise test

In the aerobic GXT, POpeak_meas_ was overestimated by 10% and compared to the WanT; more individuals deviated from the 20% boundaries (Fig 4). Six out of 17 participants showed a non-valid GXT protocol, based on test duration. Hence P30 is not considered to be a very accurate estimator of POpeak in the current group of able-bodied individuals. This is also clarified in the regression equation based on our able-bodied population, with P30 as independent variable to estimate POpeak. It showed a lower explained variance, compared to earlier studies that included wheelchair-dependent individuals (R^2^ = 0.67 vs. R^2^ = 0.81 [10] and R^2^ = 0.77 [28]; Table 2). Wheelchair-dependent individuals commonly rely on the limited active upper-body muscle mass during any wheelchair task in sports, as well as in daily life, anaerobically and aerobically [32]. By contrast, able-bodied participants can increase working muscle mass completely differently (e.g. by using their legs and/or trunk) and are not exclusively dependent on their upper-body muscle mass, which clarifies the lower explained variance [33].

Another reason for the earlier-than-expected termination of the GXT can be attributed to RPE. The current study showed higher RPE_p_ than RPE_c_ scores: 9 ± 1 vs 7 ± 1. This concords with previous research in untrained able-bodied participants performing arm-crank [34], hand-cycle [35] and wheelchair propulsion [36–39]. Different results were observed when compared to elite wheelchair rugby athletes, where no differences between RPE_p_ and RPE_c_ after a GXT were identified [39]. Wheelchair propulsion is a rather specific (and straining) movement [40], and because able-bodied participants are not used to this movement they mainly gave up because of local, not cardiorespiratory fatigue. While wheelchair athletes are trained in wheelchair propulsion, they might go on until cardiorespiratory fatigue (i.e. a higher RPE_c_) during the GXT, so overestimation is not expected to occur. This must be corroborated in future research.

### 4.3 Limitations and future research

The recruitment of able-bodied participants who were inexperienced in wheelchair propulsion and arm exercise limits generalizability of the results. Able-bodied participants were included as a first exploration to investigate the potential of this protocol design on a wheelchair roller ergometer before being applied to an athletic wheelchair population. Although the current paper shows good results for the WAnT, future research should investigate whether the same results are obtained in athletic wheelchair populations and whether the sprint test could also estimate WAnT resistance. For the GXT, based on the possibly stronger associations among these capacities in wheelchair athletes and the expected differences in RPE, the GXT protocol settings must first be evaluated in wheelchair athletes before considering alterations to the standardized and individualized test protocol.

It must be noted that no spirometry was adopted in the current study; no conclusions can be drawn on physiological processes during graded exercise testing (e.g. peak oxygen uptake (VO_2_peak), respiratory exchange ratio) [20]. This is for the future. The current study simply focused on defining valid resistance settings to attain exhaustion between 8 and 12 min, meeting an estimated peak aerobic power output ± 20%, it was not aimed at a valid VO_2_peak [8]. To at least evaluate whether the participants were close to their true maximal aerobic capacity, secondary criteria were set based on HR and RPE [20], both of which have limitations yet worked fairly well. There is variability in the HR_peak_ of individuals, and RPE remains a subjective measure that requires practice [41, 42]. Together with RPE_p_ as limiting factor in the GXT, this may explain why three participants had a valid test protocol based on test duration, but did not meet the HR_peak_ and RPE criteria (Table 1). We encourage adopting spirometry for a more objective insight into the attainment of a valid VO2peak in future studies.

Two locations with two test leaders and two wheelchairs were used in this study. The results per location are also visualized in Fig 4, and no clear difference between the two locations can be seen. We tried to rule out excessive variation, yet we may have induced some variability between the two groups because of factors like a different wheelchair set-up or different verbal encouragement from the test leaders [43, 44]. Besides, we tested half of the participants in the morning and the other half in the afternoon, which might have caused variation between the two groups due to the circadian effect (time of day) [45]. However, the focus of this study was on evaluating the test protocol based on individual associations among wheelchair-specific isometric strength and anaerobic and aerobic capacity. Since the wheelchair, test leader and time of day did not change within individuals, this probably had a limited influence on the results.

### 4.4 Practical implications

Implementing the methods explored in this study can have a positive impact on future wheelchair-specific exercise capacity testing. Literature evidences a large variation in WAnT and GXT test protocols [9]; the currently studied standardized and individualized resistance settings can produce more uniform protocols and settings. This is especially needed in the diverse athletic and/or patient populations, and will enhance comparisons between different studies as well as among athletes or patients over time [9].

When an instrumented wheelchair roller ergometer is not available, treadmills can be an alternative, although they do not allow isometric strength, sprint and WAnT testing [16]. However, an isometric strength test could be performed in an alternative set-up [12] and can provide an indication of POpeak in the GXT [10]. In this way, it remains possible to utilize this standardized and individualized protocol design.

## 5 Conclusion

The current study evaluated a test protocol with standardized and individualized resistance settings to measure wheelchair-specific anaerobic and aerobic exercise capacity in healthy novices on an instrumented wheelchair ergometer. The isometric strength test can be used to individually scale the test protocol for the WAnT in able-bodied participants. However, because of slipping, stronger participants might have to use an alternative test, e.g. the 10 s sprint test, to estimate anaerobic power and determine individual WAnT resistance. The WAnT outcomes scaled the protocol for the GXT less accurately, resulting in partly non-valid GXT results; this could be due to the inexperienced able-bodied group. Literature shows a large variation in WAnT and GXT test protocols. The currently evaluated standardized and individualized wheelchair-specific exercise capacity protocol could provide a more uniform design of test protocols in wheelchair athletes. Its appropriate use in paralympic wheelchair sports practice must first be further evaluated in well-controlled research settings among wheelchair athletes.
